# Low‐dose hilar and mediastinal stereotactic body radiation therapy for non‐small cell lung cancer: Analysis of outcomes in patients receiving one or multiple courses of treatment

**DOI:** 10.1111/1759-7714.13501

**Published:** 2020-05-29

**Authors:** Roman O. Kowalchuk, Michael R. Waters, Martin Richardson, Kelly Spencer, James M. Larner, Charles R. Kersh

**Affiliations:** ^1^ University of Virginia / Riverside Radiosurgery Center Newport News Virginia USA; ^2^ University of Virginia Department of Radiation Oncology Charlottesville Virginia USA

**Keywords:** Carcinoma, non‐small cell lung, dose fractionation, radiation, lung neoplasms, patient selection, radiosurgery

## Abstract

**Background:**

This study reports the outcomes of a single institutional experience treating non‐small cell lung cancer (NSCLC) involving the pulmonary hilum with low‐dose stereotactic body radiation therapy (SBRT). The authors also present a series of repeat hilar SBRT.

**Methods:**

Inclusion criteria required treatment with SBRT for NSCLC involving regional lymph nodes of the: (i) hilum, (ii) mediastinum, (iii) aortopulmonary window (station 5), or (iv) mainstem bronchus. At least one clinical follow‐up with imaging was required, unless the patient had a prior documented death from cancer.

**Results:**

A total of 32 patients with 44 treatments were included, and 37 treatments targeted the hilum directly, with seven concerning the mediastinum, AP window, or mainstem bronchus. Median dose was 28 Gy in four fractions with once‐weekly fractionation. At a median clinical follow‐up of 23 months, local control was 64%. Median overall survival was 24 months, and median progression‐free survival was 15 months. A total of 48% of treatments resulted in complete radiographic response on last imaging follow‐up, and no cases of grade ≥ 3 toxicity were reported. For repeat SBRT (after prior hilar SBRT), local control was 92%. Median overall survival was 20 months, and median progression‐free survival was 19 months. Complete radiographic response was noted after 58% of treatments, with 0 instances of progressive response and no reported side effects.

**Conclusions:**

Low‐dose hilar SBRT was efficacious and well‐tolerated, with impressive overall survival and no grade ≥ 3 toxicity. Repeat treatments with SBRT were feasible and effective, demonstrating overall survival, local control, and toxicity comparable to primary treatments.

**Key points:**

**Significant findings of the study**

- Low‐dose hilar SBRT was efficacious and well‐tolerated
- Repeated treatments with SBRT demonstrated encouraging results, comparable to primary treatments

**What this study adds**

- This study contributes to the small body of literature concerning hilar SBRT
- Repeat hilar SBRT was safe and feasible
- Toxicity was minimal with low‐dose SBRT
- Once‐weekly fractionation may have contributed to low rate of side effects.

## Introduction

Lung cancer remains the leading cause of cancer mortality in men and women in the US and worldwide.^1^ Treatment options are varied, including surgery, chemotherapy, radiotherapy, and immunotherapy. As clinical understanding of biomarker testing has progressed, options for therapy have become increasingly personalized.^2^, ^3^ Stereotactic body radiation therapy (SBRT), also known as stereotactic ablative radiotherapy (SABR), is an effective treatment modality for early stage lung cancer or metastases to the lungs that involves delivering high doses of radiation to the tumor in relative few fractions (generally 3–5).^4^, ^5^, ^6^, ^7^ High treatment doses, with biologically effective dose (BED) ≥100 Gy, have consistently demonstrated impressive rates of local control.^8^

A variety of SBRT treatment regimens have demonstrated acceptable tumor control without severe toxicity, but centrally located tumors represent a higher‐risk tumor location and predispose patients to a unique toxicity profile, including radiation pneumonitis and pulmonary hemorrhage.^9^, ^10^ A commonly used definition for a central tumor is a lesion within 2 cm in all directions of any mediastinal critical structure, and this definition was utilized in this analysis. Generally, caution has been advised for tumors within 2 cm of the proximal bronchial tree.^11^ For centrally‐located lung tumors, larger tumor diameters have been correlated with increased rates of grade ≥ 3 toxicity.^12^ Additionally, tumors <1 cm from the proximal bronchial tree have been correlated with a higher risk of noncancer death and grade ≥ 3 toxicity.^13^, ^14^ A recent retrospective study of 108 patients who received SBRT for central lung tumors found that of the 18 patients with tumors abutting the proximal bronchial tree, four suffered from grade 5 toxicity.^14^ Concern regarding these potential side effects has resulted in the identification of the maximum point dose to the mainstem bronchus, mean lung dose, and V20 as objective treatment components to minimize so as to decrease the risk of toxicity.^15^, ^16^, ^17^ Many studies, however, have reported treatment efficacy with tolerable toxicity in patients with centrally‐located tumors, including the RTOG 0813 trial, in which a local control rate of 87.9% was shown.^18^, ^19^, ^20^, ^21^, ^22^, ^23^, ^24^

The various subgroup classifications within treatments for centrally‐located tumors have resulted in a new distinction of “ultracentral” tumors. Chaudhuri *et al*. defined “ultracentral” tumors as those with GTV directly abutting the central airway.^25^ In another study, “ultracentral” lung tumors were defined as those with a planning target volume (PTV) overlapping the trachea or main bronchi. Toxicity results demonstrated high rates of fatal pulmonary hemorrhage (15%) and any grade ≥ 3 toxicity in 38% of patients.^26^ On the other hand, Chang *et al*. reported no significant differences between central and ultracentral lung tumors regarding overall survival, local failure, or grade ≥ 3 toxicity.^27^

Within the context of central and ultracentral tumors, the concept of hilar lung involvement also requires exploration. In fact, tumor extension into the hilum has been highly related with prognosis. The hilum can be involved through direct tumor extension, lymph node spread from primary non‐small cell lung cancer (NSCLC), or via metastatic spread of a different primary malignancy.^28^ New imaging techniques, such as 3D‐dynamic MRI, have been increasingly utilized to better characterize hilar adenopathy.^29^, ^30^ These modalities assist in choosing between treatment options ranging from chemotherapy, conventional radiotherapy, SBRT, surgery, or a combination of treatments.^31^ Conventional radiotherapy can successfully salvage lymph node relapses after SBRT or surgery, with five‐year local control of 58% for patients after SBRT.^32^, ^33^ In the case of lymphadenopathy, the standard therapy consists of combination chemotherapy and radiotherapy. While SBRT is not contraindicated for hilar or mediastinal structures, data regarding its use is extremely limited.^34^, ^35^, ^36^ For instance, Horne *et al*. noted two year local control of 87.7%, and acute grade ≥ 3 toxicity was seen in only three of 40 patients, along with late grade ≥ 3 morbidity in one patient.^15^ The purpose of this study is to report the results of a single institutional experience of treating locally advanced and metastatic NSCLC with SBRT to hilar and mediastinal lymph nodes. We will report overall survival, progression‐free survival, local control, and toxicity and describe a series of cases regarding repeat hilar SBRT after prior therapy with hilar SBRT.

## Methods

### Patient cohort

All patients treated with SBRT for hilar involvement of NSCLC from January 2007–November 2018 at a single, high‐volume radiosurgery center were considered for inclusion in the study. The study population considered locally advanced and metastatic NSCLC involving the pulmonary regional lymph nodes and treated with SBRT for lymph node disease. These patients often presented for SBRT after another form of treatment, but most presented with systemic disease control. Due to the high rates of previous treatment and the toxicity risk inherent to the central location of the targeted lymph nodes, low dose SBRT was often employed. Inclusion criteria required treatment with SBRT for NSCLC involving regional lymph nodes of the: (i) hilum, (ii) mediastinum, (iii) aortopulmonary (AP) window (station 5), or (iv) mainstem bronchus. Exclusion criteria included treatments with at least 10 fractions of radiotherapy or a different primary tumor (aside from NSCLC). At least one clinical follow‐up with imaging was required for inclusion, unless the patient had a documented death from the cancer prior to that time. In this way, patients lost to follow‐up would not be included, but the survival analysis would still be an accurate representation of the disease process. All patients with primary small cell lung cancer were also excluded from the study. Patients were not excluded from the study if they lacked a biopsy, provided that (i) all such patients were empirically treated for NSCLC, and (ii) no evidence of a different underlying disease pathology was noted on retrospective review. For inclusion in the repeat SBRT cohort, patients were required to have had multiple courses of SBRT to the pulmonary hilar region, and only the repeat treatment was considered. That is, if a patient received two SBRT treatments to the hilum, only the second treatment was included; if a patient received three SBRT treatments to the hilum, the latter two were included.

Patient‐specific characteristics (eg, COPD status, performance status, and whether or not a biopsy was obtained) were recorded by a single investigator to minimize bias. All prior treatment for NSCLC was recorded, including chemotherapy, surgery, or radiation therapy (including radiotherapy to the thoracic cavity for reasons other than NSCLC). Key dosimetric data included the dose‐fractionation scheme, target location, gross tumor volume (GTV), PTV, and the mean, maximum, and minimum dose per fraction. An alpha/beta ratio of 10.0 Gy was used for calculations of the BED, using the formula BED = total dose * (1 + dose per fraction/alpha/beta ratio). The study was exempt by the institutional review board.

### Radiation therapy

Where applicable, previous radiation therapy treatment planning files, including DICOM‐RT dose files, were obtained and imported into the treatment planning system for evaluation of prior dose to organs‐at‐risk. Patients were immobilized using a full‐body vacuum bag system for position stabilization and consistency. Serial CT scans were taken (free‐breathing, inhale, exhale) for treatment planning purposes and to assess the motion of the target during the breath cycle and generate an internal‐target‐volume (ITV) margin around the GTV. Dose was prescribed to the PTV which is the ITV + 3–5 mm of margin to account for uncertainties in imaging and localization. In general, a 3D conformal treatment planning approach with noncoplanar gantry angles was used to minimize dosimetric overlap of entrance and exit portals; intensity‐modulated radiotherapy (IMRT) and volumetric‐modulated arc therapy (VMAT) were considered. Dosimetry from previous radiation therapy was assessed, and attempts to avoid significant dosimetric overlap in critical organs were made. SBRT was delivered using a 6 MV photon beam on a linear accelerator with a 2.5–4 mm width multileaf collimator for custom shaping of portals. On‐board cone‐beam CT (CBCT) with 4D capabilities was used prior to treatment, and a 6D robotic couch assisted in the alignment of the patient and localization of the target to the planning CT. CBCT would be repeated several times during treatment to correct any intrafraction motion of the patient or target. Dose‐fractionation schemes generally involved once weekly fractionation, in an effort to decrease side effects from therapy. Times between fractions were recorded noninclusively, such that there were six days between once‐weekly treatments.

### Patient follow‐up

The major endpoints of this analysis were local control and toxicity. Radiographic response, overall survival, progression‐free survival, and local‐progression free survival were also recorded as important secondary endpoints. Patient follow‐up was conducted by radiation oncology, hematology‐oncology, and pulmonology. Overall survival was recorded from the completion of SBRT to the last documented interaction with a healthcare provider. Imaging follow‐up was conducted at regular two to three month intervals with CT and/or PET imaging to determine treatment response. Radiographic response was delineated as progressive, stable, improved, or complete. Local, regional, and distant failure dates were all recorded as the date of the imaging on which the treatment failure was observed. Corresponding times to local, regional, distant, or any failure were calculated. Finally, the need for any subsequent therapy was included as a clinical outcome and distinguished as additional chemotherapy, surgery, or radiotherapy (SBRT, external beam radiation therapy (EBRT), or gamma knife (GK)). Acute and chronic toxicity data, including any cases of radiation pneumonitis or hemorrhage, were graded in accordance with the Common Terminology Criteria for Adverse Events (CTCAE) v5.0. Ninety days was used as the cutoff for distinguishing acute from chronic toxicity.

### Statistical analysis and predictive factors

Statistical analysis was conducted for each treated lesion to analyze local control and radiographic response, and survival analysis was performed for each patient to assess for overall survival and progression‐free survival. The patients who underwent repeat SBRT to a hilar target were also separated to analyze these outcomes for that subgroup, specifically. To further assess predictive factors, Cox proportional hazards regression analysis was utilized. A threshold *P*‐value of 0.05 was used to denote statistical significance. The Kaplan‐Meier method was also incorporated in order to accurately demonstrate the overall survival, progression‐free survival, and local control, including distinctions between the endpoints of the different subgroups of the cohort.

## Results

### Patient characteristics

A total of 32 unique patients met the inclusion criteria for the study, and there were 44 distinct treatments for these patients. Predominantly, the patients had Karnofsky performance status (KPS) > 70 (77%). While most patients had a biopsy obtained (64%), 36% of patients did not. In most cases, a biopsy was not performed due to concerns regarding the patient's poor pulmonary function and/or inability to tolerate the procedure, thus generally indicating worse performance status or more advanced disease. A total of 57% of patients presented for treatment at stage 3, and 20% had stage 4 NSCLC. For stage ≥3 patients, SBRT was generally chosen secondary to the patients not being optimal candidates for multimodal therapy. Specifically, the median age of the patients in this study was 76.13 years, and a majority had chronic obstructive pulmonary disease (COPD) (61%). Even so, systemic disease was deemed to be controlled in 82% of cases. Finally, any prior treatments were recorded, and the times from primary diagnosis and prior treatment to the new SBRT therapy were incorporated into Table 1. Of note, 50% of patients had received prior chemotherapy while 75% had received prior radiotherapy of some kind. A total of 68% of patients presented after local failure of the primary treatment, which occurred a median 19 months prior to SBRT.

**Table 1:** Patient demographics are shown

	Number	Fraction
Patients	32	
Treated lesions	44	
Male	14	
Female	18	
Median age	76.13	
KPS >70	34	0.77
KPS = 70	10	0.23
COPD	27	0.61
Biopsy obtained	28	0.64
Adenocarcinoma	15	0.34
Squamous cell carcinoma	10	0.23
Unknown histology	19	0.43
Systemic disease controlled	36	0.82
Stage 2	9	0.20
Stage 3	25	0.57
Stage 4	9	0.20
Stage NA	1	0.02
Synchronous lesions	20	0.45
Prior chemotherapy	22	0.50
Prior surgery	9	0.20
Prior radiotherapy	33	0.75
Prior EBRT	14	0.32
Prior SBRT	15	0.34
Both EBRT and SBRT	4	0.09
Local failure after primary treatment	30	0.68
Primary diagnosis to new SBRT (months)	25.03	
Prior treatment to new SBRT (months)	18.89	

### Radiation therapy

Table 2 presents the data concerning the patients' radiotherapy. No patients received concurrent chemotherapy. A total of 37 of 44 (84%) treatments targeted the hilum directly, with the remaining seven concerning the mediastinum, AP window, or mainstem bronchus. Median dose was 28 Gy in four fractions, with a median BED of 47.6 Gy. Patients had a median 5.25 days between fractions. Because there were no exclusion criteria based on treatment volume, GTV and PTV both had wide ranges. Median GTV was 8.21 cc (range: 0.99–159.2 cc), and the median PTV was 15.15 cc (range: 1.28–269.6 cc).

**Table 2:** Radiation therapy details for the entire cohort are demonstrated, including dosimetric factors and the target

	Number	Fraction
Concurrent chemotherapy	0	0.00
Right hilum	22	0.50
Left hilum	15	0.34
Mediastinum	3	0.07
AP window	2	0.05
Mainstem bronchus	2	0.05
Median dose (Gy)	28 (15–50)	
Median fractions	4 (2–5)	
Median BED (Gy)	47.6 (22.5–112.5)	
Median time between fractions (days)	5.25 (2.80–9.33)	
Median GTV (cc)	8.21 (0.99–159.2)	
Median PTV (cc)	15.15 (1.28–269.6)	
Median PTV mean dose/fraction (cGy)	731 (432–1375)	
Median PTV max dose/fraction (cGy)	765 (455–1418)	
Median PTV min dose/fraction (cGy)	608 (274–1188)	

### Patient outcomes

The key endpoints of the study are tabulated in Table 3. There was a median clinical follow‐up of 23 months. Overall, local control was 64%, with 16 total instances of local failure at a median 15 months after SBRT. There were also nine cases of regional failure (20%) and six distant failures (14%).

**Table 3:** Patient outcomes are presented, including local control, overall survival (OS), progression‐free survival (PFS), local progression‐free survival (LPFS), and radiographic response

	Number	Fractions
Median follow‐up (months)	22.70 (0–97.87)	
Local control	28	0.64
Local failure	16	0.36
Median time to local failure (months)	15.34 (3.71–26.94)	
Regional failure	9	0.20
Median time to regional failure (months)	8.97 (2.37–26.94)	
Distant failure	6	0.14
Median time to distant failure (months)	13.54 (5.12–25.33)	
Disease‐free at last follow‐up	19	0.43
Median imaging follow‐up (months)	16.69 (0.59–93.29)	
Follow‐up with CT	29	0.66
Follow‐up with PET/CT	15	0.34
Patients deceased	23	0.72
Patients alive	9	0.28
Median OS (months)	23.51 (0.69–100.76)	
Median PFS (months)	15.34 (0.69–75.93)	
Median LPFS (months)	16.15 (0.69–75.93)	
Subsequent treatment	19	0.59
Subsequent SBRT	17	0.53
Radiographic response		
Complete	21	0.48
Improved	6	0.14
Stable	14	0.32
Progressive	3	0.07
Grade < 3 toxicity	6	0.14
Grade ≥ 3 toxicity	0	0
Radiation pneumonitis	0	0

**Figure 1:**
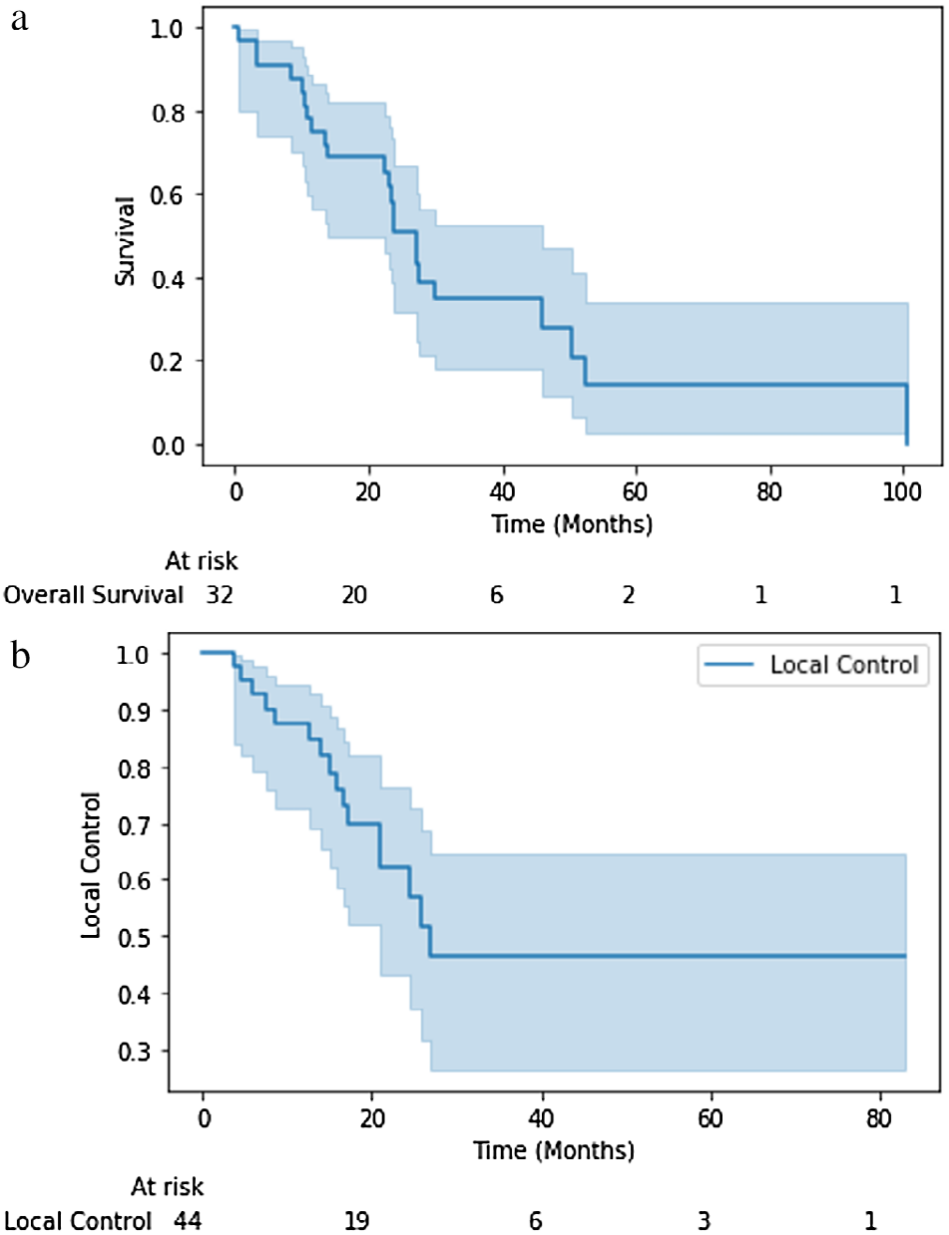
Outcomes for the entire cohort are presented, including (**a**) overall survival; and (**b**) local control. (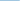) Overall survival, (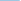) Local control.

**Figure 2:**
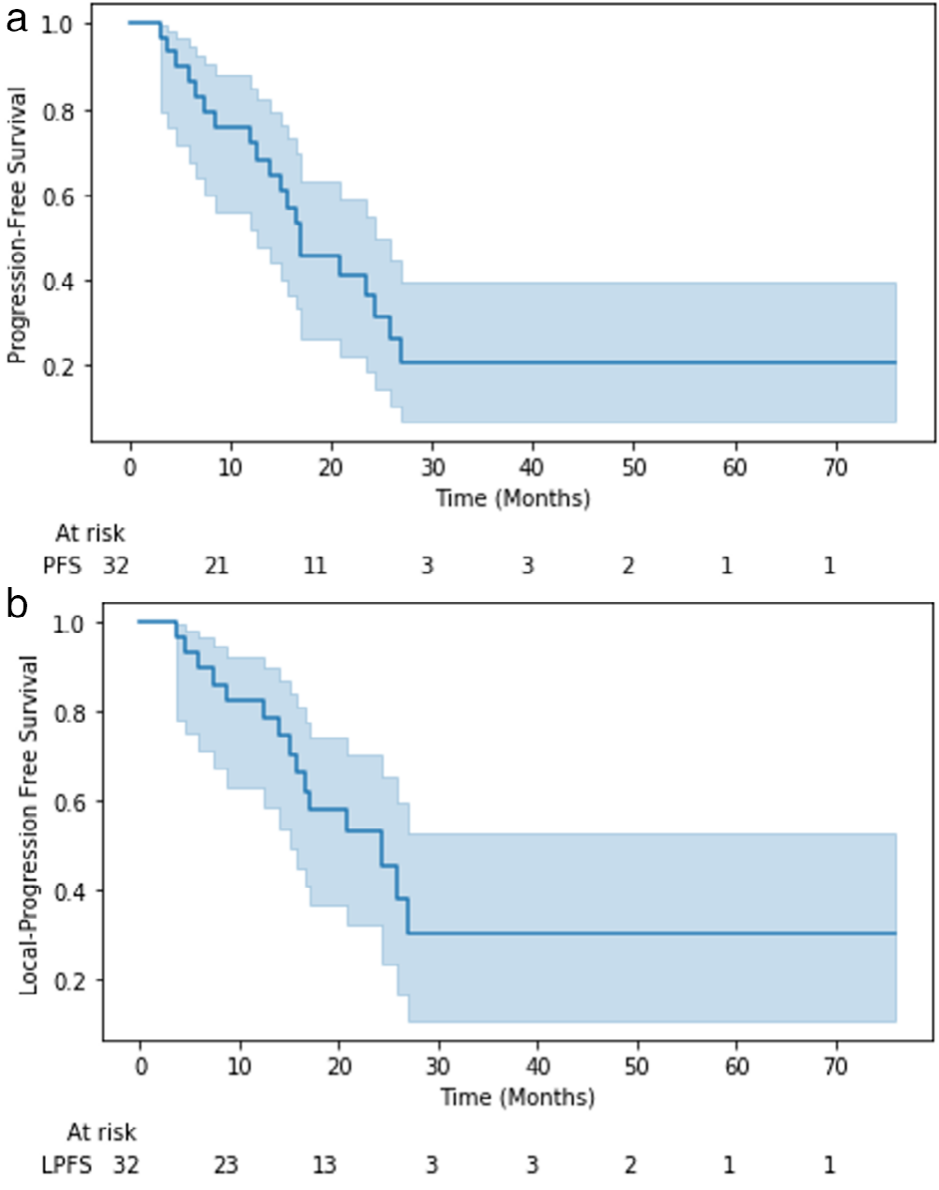
(**a**) Progression‐free survival (PFS); and (**b**) local progression‐free survival (LPFS) are illustrated using the Kaplan‐Meier method. (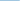) PFS, (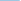) LPFS.

There were 9 patients (28.1%) still alive at the time of review, with a median overall survival of 24 months (Fig 1). Median progression‐free survival was 15 months, and median local progression‐free survival was 16 months (Fig 2). A total of 19 patients (59%) required additional therapy after the completion of SBRT, with 17 (53%) receiving additional SBRT of some kind. Twelve of these treatments involved repeat hilar SBRT while three involved distant metastases of NSCLC. The remaining two SBRT treatments were for different primary cancers. Two patients also received treatment with Gamma Knife. Only one patient received additional systemic chemotherapy. In this case, three cycles of carboplatin, pemetrexed, and bevacizumab were given. Radiographic response was graded a scale of complete response (48%), improved (14%), stable (32%), and progressive (7%) at a median imaging follow‐up of 17 months.

### Predictive factors

On univariate and Kaplan‐Meier analyses, no factors were found to be predictive of local control. Factors tested included: gender, COPD status, whether or not a biopsy was obtained, histology, prior treatments, stage and systemic disease status, time from diagnosis or prior treatment to SBRT, and the dosimetric factors of dose, BED, GTV, and PTV. The only factor predictive of complete radiographic response was the lack of prior surgical intervention. Dosimetric factors, including dose, BED, GTV, and PTV, were not predictive of this outcome, nor were the times from primary diagnosis or prior treatment to presentation for SBRT. Univariate analysis also failed to demonstrate any predictive factors for overall survival at 24 months, testing the same factors as above. Multivariate analysis was conducted for local control and overall survival; though multiple factors trended towards statistical significance (e.g., having had a biopsy trended towards improved local control), no factors tested explicitly showed a *P*‐value <0.05.

### Toxicity

The treatment was very well tolerated, with no cases of grade ≥ 3 toxicity. There were also no cases of radiation pneumonitis reported in the entire patient cohort. Six total treatments resulted in grade 1 or 2 toxicity (14%), with only one reported grade 2 toxicity of shortness of breath. The five cases of grade 1 toxicity consisted of shortness of breath (3) and fatigue (2).

### Repeat SBRT patients

The unique patient subgroup which received multiple SBRT treatments to the pulmonary hilum was also considered separately (Table 4). There were eight such patients who received at least one repeat treatment and 12 total treatments of this kind. A total of 11 treatments involved local failure after the previous SBRT course, and one treatment consisted of two synchronous lesions in the left hilum treated with two separate courses of SBRT. A total of 10 targeted the hilum directly, with one each to the mediastinum and the AP window. Median dose was 22 Gy in four fractions, with a median BED of 34.2 Gy. Median GTV and PTV were 9.22 cc and 10.35 cc, respectively.

**Table 4:** A description of the repeat SBRT treatments is presented, along with treatment outcomes of local control, overall survival (OS), and progression‐free survival (PFS)

	Number	Fraction
Patients	8	
Treatments	12	
Right hilum	6	0.50
Left hilum	4	0.33
Mediastinum	1	0.08
AP window	1	0.08
Mainstem bronchus	0	0.00
Median dose (Gy)	22 (15–28)	
Median fractions	4 (3–4)	
Median BED (Gy)	34.2 (22.5–47.6)	
Median time between fractions (days)	5.25 (3.67–9.33)	
Median GTV (cc)	9.22 (1.28–46.8)	
Median PTV (cc)	10.35 (1.28–116)	
Median PTV mean dose/fraction (cGy)	631 (514–745)	
Median PTV max dose/fraction (cGy)	650.5 (521–769)	
Median PTV min dose/fraction (cGy)	509 (389–693)	
Local control	11	0.92
Local failure	1	0.08
Regional failure	3	0.25
Distant failure	2	0.17
Disease‐free at last follow‐up	8	0.67
Patients deceased	5	0.63
Patients alive	3	0.38
Median OS (months)	19.63 (6.70–83.06)	
Median PFS (months)	19.07 (2.37–83.06)	
Radiographic response		
Complete	7	0.58
Improved	2	0.17
Stable	3	0.25
Progressive	0	0.00

### Repeat SBRT patient outcomes

**Figure 3:**
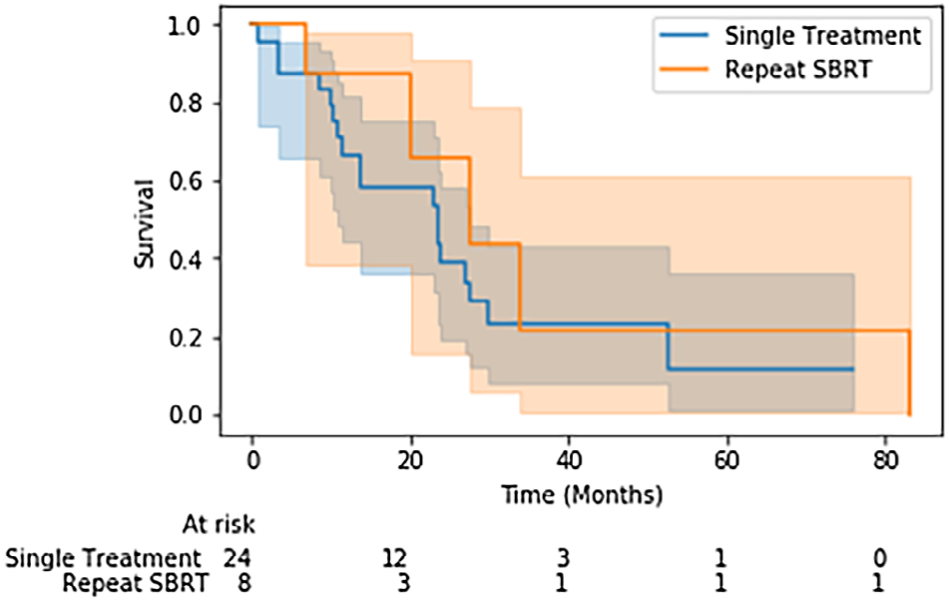
Overall survival for one versus multiple treatments is presented, and there was no statistically significant difference between the two cohorts. (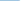) Single treatment, (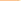) Repeat SBRT.

Local control was 92%, with only one instance of local failure. There were three cases of regional failure (25%) and two distant failures (17%). After eight treatments (67%), disease‐free status at last follow‐up was obtained. Three of eight patients (38%) were still alive at the time of chart review, with a median overall survival of 20 months and median progression‐free survival of 19 months. The repeat SBRT cohort demonstrated comparable levels of stage ≥3 disease, compared to primary SBRT (83% vs. 77%). There was no difference between the overall survivals of primary treatment and repeat treatment on univariate analysis. Kaplan‐Meier analysis in Fig 3 also corroborated this finding that there was no difference in survival between patients requiring multiple treatments versus only a single course of SBRT. For repeat SBRT, radiographic response demonstrated complete response after 58% of treatments, with 0 instances of progressive response. None of the eight patients suffered from any side effects during the course of the repeat treatments.

## Discussion

This study presents outcomes from low‐dose hilar SBRT and includes a subgroup of patients treated with repeat hilar SBRT. Considering this patient cohort as a whole, the treatment was efficacious. Impressive overall survival (24 months) and progression‐free survival (15 months) were reported, and the local control rate was 64%. Other studies of central thoracic SBRT have reported higher local control rates of upwards of 80%^12^, ^19^; however, controversy surrounding central lung SBRT has revolved primarily around toxicity, and this patient population demonstrated no grade ≥ 3 toxicity.^9^, ^11^, ^14^, ^26^ Although some reports have correlated greater tumor diameter with increased toxicity in the setting of central lung tumors, this analysis exhibited low toxicity without excluding patients based on tumor size.^12^ Additionally, GTV and PTV were not correlated with local failure. In fact, not only was toxicity minimal in the patient group as a whole, but repeat SBRT treatments were also well‐tolerated, with impressive local control of 92% and no treatment toxicity. Repeat treatments in this study were conducted with quite low doses, with only a median BED of 34.2 Gy. This low dose is seemingly in contrast with other findings regarding the benefits of delivering higher doses of radiation to the tumor target.^8^ Even so, this additional dose delivery was effective and well‐tolerated. Overall survival in this cohort of patients presenting after local failure of primary SBRT was no different than for patients who initially maintained local control, further indicating a role for repeat therapy. Other outcomes, such as radiographic response and progression‐free survival, were comparable or even improved in patients after the second course of treatment. Though there is a paucity of high‐quality data, these results strongly point towards consideration of repeat SBRT for hilar targets in NSCLC, particularly with low doses to minimize toxicity.^37^

Within the context of the broader question concerning the safety of SBRT to the central thoracic structures, literature regarding treatment of the pulmonary hilum itself is quite limited. Though a questionnaire of 26 centers found that adjacency to hilar/mediastinal structures was not considered to be a contraindication for SBRT for most providers, there is little data on the subject.^34^, ^35^ In 2010, Oshiro *et al*. published findings from 21 patients who underwent SBRT for lung tumors within 2 cm of a major bronchus and concluded that SBRT in the pulmonary hilar region may be tolerable if irradiated volumes are carefully selected.^36^ Unfortunately, their study population was defined using the common definition of a central lung tumor (within 2 cm of a proximal bronchus), making their conclusion concerning the hilar region more difficult to interpret. The most similar study to the present analysis was conducted by Horne *et al*. in 2018, which was at the time the largest single institutional series of thoracic nodal SBRT.^15^ They considered a group of 40 patients with primary or oligorecurrent hilar/mediastinal NSCLC treated for targets involving the AP window (40%), hilum (25%), lower paratracheal (20%), subcarinal (10%), and prevascular (5%) regions. The median dose was 48 Gy in four fractions, and they reported median overall survival and progression‐free survival of 22.7 and 13.1 months, respectively. Local control was 87.7% and not different between hilar and mediastinal targets, but hilar targets demonstrated improved progression‐free survival. They noted acute grade ≥ 3 toxicity in 7.5% of patients and one case of late grade 3+ morbidity.

Three critical aspects of the patient population serve to distinguish this study from those findings: (i) this analysis consists of a much higher fraction of hilar SBRT (84%); (ii) the dose provided in the population of Horne *et al*. was much higher than the dose provided in this analysis (median 28 Gy in four fractions); and (iii) this patient cohort included 12 repeat SBRT hilar treatments not included in the other study. Overall, they demonstrated comparable overall survival (22.7 vs. 24 months) but higher local control (87.7% vs. 64%). It is possible that this improved local control is related to the higher dose delivered in their study, or it could be related to the more diverse targets included in their analysis. Their finding that there was no difference between the local control rates of the hilar and mediastinal targets would further support the first hypothesis; however, their study may have been underpowered to demonstrate any such difference since it only included 10 patients with hilar SBRT targets. Further, it is possible that the higher dose initially may have contributed to their higher local control rate while the lower dose used in this study may have allowed for repeat therapy. At least in this analysis, the latter strategy provided comparable survival, without any grade ≥ 3 toxicity. In this study, once‐weekly fractionation was employed in an effort to reduce toxicity. This approach merits further exploration in this setting because it does extend the time over which the therapeutic dose is delivered and increases the overall time to treatment completion. On the other hand, it allows for increased time for reoxygenation, which could increase the efficacy of treatment by reducing the fraction of surviving hypoxic cells.^38^, ^39^

Limitations of this study primarily relate to the fact that SBRT for hilar spread of NSCLC is still a relatively new treatment modality only in use in the last 10–20 years. Greater patient numbers might have allowed for predictive factors for local control and overall survival to be determined, and additional repeat SBRT patients may have revealed some differences between the patient subgroups. The retrospective nature of the analysis also carries inherent limitations. Finally, an important future direction of study involves the comparison of SBRT to different treatment options and techniques for the treatment of mediastinal nodal failure. The use of simultaneous integrated protection for organs at risk has offered the potential for dose escalation.^40^, ^41^ Simultaneous integrated boost for mediastinal nodal recurrence also demonstrated feasibility and safety in a recent pilot study.^42^ Since the present analysis involved low dose SBRT, the incorporation of such techniques could notably improve outcomes.

In conclusion, this study demonstrates treatment efficacy and safety for low‐dose hilar SBRT, with impressive overall survival and no grade ≥ 3 toxicity despite the high‐risk anatomic location. This analysis also demonstrates encouraging results regarding low dose, repeat SBRT treatment. Repeat treatments were feasible and effective, demonstrating overall survival, local control, and toxicity comparable to primary treatments. This study shows that SBRT should be considered as a primary or salvage treatment for hilar spread of NSCLC.

## Disclosure

The wife of Dr Kowalchuk works for GE Healthcare as a senior technical product manager. The rest of the authors confirm that there are no potential conflicts of interest, including financial interests, relationships, and affiliations relevant to the subject of the manuscript.
